# Pathogenesis, diagnosis and management of primary melanoma of the colon

**DOI:** 10.1186/1477-7819-9-14

**Published:** 2011-02-01

**Authors:** Umair Khalid, Taimur Saleem, Ayesha Mallick Imam, Muhammad Rizwan Khan

**Affiliations:** 1 Medical College, Aga Khan University, Stadium Road, Karachi 74800, Pakistan; 2 Section of General Surgery, Department of Surgery, Aga Khan University, Stadium Road, Karachi 74800, Pakistan

## Abstract

**Background:**

Melanomas within the alimentary tract are usually metastatic in origin. On the other hand, primary melanomas of the gastrointestinal tract are relatively uncommon. There are several published reports of melanomas occurring in the esophagus, stomach, small bowel, and anorectum. The occurrence of primary melanoma of the colon has, however, only been rarely reported. The optimum modus operandi for the management of primary colonic melanoma remains nebulous due to the limited number of reports in literature.

**Methods:**

A comprehensive search of Medline, Cochrane and Highwire was performed using the following keywords: 'melanoma', 'malignant melanoma', 'primary melanoma', 'colon', 'gastrointestinal tract', 'alimentary tract', 'digestive tract', and 'large bowel'. All patients with primary melanoma localized to the colon were included in the review. Patients with metastatic melanomas to the gastrointestinal (GI) tract and primary melanomas localized to the GI tract in anatomic locations other than colon were excluded.

**Results:**

There have been only 12 reported cases of primary melanoma of the colon to date. The average age of patients on presentation was 60.4 years without any significant gender predilection. Right colon (33%) and cecum (33%) were the most common sites for the occurrence of primary colonic melanoma while abdominal pain (58%) and weight loss (50%) were the most common presenting complaints. Colonoscopy is the most reliable diagnostic investigation and offers the additional advantage of obtaining tissue for diagnosis. S-100 and HMB-45 are highly sensitive and specific for the diagnosis of this malignancy. For primary colonic melanomas that have not metastasized to any distant parts of the body, surgical resection with wide margins appears to be the treatment of choice. Although the management was individualized in every case, most of the authors preferred traditional hemicolectomy as the favored surgical approach. Chemotherapeutic agents including interferons, cytokines, biological agents and radiation therapy for brain metastases have been reported as adjuvant and palliative options while considering malignant melanomas in general. The average recurrence-free interval was 2.59 years. Nine of the 12 reports documented follow-up in their patients. Two of these 9 (22.2%) patients died.

**Conclusions:**

Primary melanoma of the colon is a rare clinical entity. Whenever a seemingly primary melanoma is detected in an atypical location such as the colon, it is prudent to conduct a thorough clinical investigation to consider the possibility of metastatic disease. Further studies are needed to document the long term follow-up, survival advantage and safety of the management approaches employed in patients with primary colonic melanoma. Based on current data, surgical resection appears to be appropriate management for primary colonic melanomas; unless the disease has metastasized to distant sites where surgery may have a limited palliative role.

## Background

Melanomas within the alimentary tract are usually metastatic in origin, with primary melanomas being relatively uncommon. The theory of primary melanoma of the gastrointestinal (GI) tract has been confirmed for lesions occurring in the esophagus, stomach, small bowel, and anorectum through several published reports, as these are the areas where melanocytes normally exist. However, the occurrence of primary melanoma in the colon is relatively rare. Nevertheless, such incidence is confounded by the embryologic absence of melanocytes in the large bowel [1]. The optimum modus operandi for the management of primary colonic melanoma also remains unclear. In this review, we have evaluated these cases and presented the collective data along with a review of the relevant literature with particular reference to the pathogenesis, diagnosis and management of primary melanoma of the colon.

## Methods

The computerized databases of Medline, Cochrane and Highwire were searched using the following keywords: 'melanoma', 'malignant melanoma', 'primary melanoma', 'colon', 'gastrointestinal tract', 'alimentary tract', 'digestive tract', and 'large bowel'. The references for each article were, in turn, also reviewed in detail for other reports of primary melanoma of the colon that may not have been detected during our initial search. All patients with primary melanoma localized to the colon were included in this review. Patients with metastatic melanomas to the gastrointestinal (GI) tract and primary melanomas localized to the GI tract in anatomic locations other than the colon were excluded. The details of these 12 cases are summarized in Table 1[1-12].

**Table 1:** Detailed description of the features of 12 reported cases of primary colonic melanoma in medical literature

#	Study	Year	Age/Sex	Site	Tumor size* (cm)	S-100	HMB-45	Melan A	Management	Outcome
1	Serin et al [1]	2010	30, M	Cecum	14	+	+	+	Right hemicolectomy and distal ileectomy	Recurrence free for at least 1 year

2	Poggi et al [2]	2000	79, M	Right colon	8	+	+	n/a	Right hemicolectomy	Recurrence free for at least 5 years

3	Avital et al ^^ [3]	2004	41, M	Right colon	6.5	-	+	+	Right hemicolectomy	Recurrence free for at least 3 years

4	Venkataraman et al [4]	2004	59, M	Left colon	n/a	+	n/a	n/a	Disseminated disease, palliative strategy employed	Not mentioned

5	McNicol et al [5]	2005	84, M	Cecum	n/a	+	+	n/a	Right hemicolectomy	Recurrence free for at least 2.5 years

6	Mori et al [6]	2006	88, F	Left colon	5	+	+	+	Partial colectomy and regional lymphadenectomy	Recurrence free for at least 3 years

7	Takahashi-Monroy et al ^^ [7]	2006	51, F	Cecum	4.2	n/a	+	+	Right hemicolectomy and resection of terminal ileum	Recurrence free for at least 1 year 8 months

8	Mandot et al [8]	2006	62, F	Right colon	n/a	+	-	n/a	Brain metastases found at the time of diagnosis; managed with steroids, temozolamide and radiation therapy	Expired within three months from the time of diagnosis; cause of death pyogenic meningitis

9	De Palma et al [9]	2006	56, F	Right colon	n/a	+	+	n/a	Right hemicolectomy	Recurrence free for at least 2 years

10	Tak et al ^ [10]	2006	72, M	Transverse colon	n/a	+	+	n/a	Patient refused any intervention	Died in 8 weeks from the time of initial presentation

11	Kenney et al [11]	2007	64,M	Transverse colon	5.5	+	-	+	Left hemicolectomy & appendectomy	Unremarkable follow up; recurrence free duration not mentioned

12	Sashiyama et al ^ [12]	2010	39, F	Cecum	2	n/a	+	n/a	Laparoscopic ileocecal resection	Follow-up not mentioned

n/a = not applicable or information not available

*greatest dimension of the resected tumor mass (only applicable to cases where surgical intervention was performed)

^ neoplams that were found to be amelanotic on gross appearance

^^ patient had obstruction secondary to intussusception.

### Epidemiology

#### a. **Types of melanoma**

Worldwide, more than 7,000 people die of malignant melanoma every year [13]. The vast majority of these cases are cutaneous melanomas. In the remaining cases, ocular melanomas are the most common type, followed by the melanomas in leptomeninges, oral cavity, nasal mucosa, pharynx, esophagus, bronchus, and vaginal or anorectal mucosa [14]. Overall, only 20% of these non-cutaneous melanomas originate in the mucosa, accounting for 3-4% of all melanomas that are diagnosed annually [2].

#### b. **Family history**

Generally, a positive family history is an established risk factor for developing melanomas. In population-based studies, 1 - 13% of cases have reported melanoma in at least one first-degree relative [15].

#### c. **Gastrointestinal involvement**

Approximately 1-4% of all patients with malignant melanoma will have clinically apparent GI tract involvement diagnosed ante-mortem, and as many as 60% of all patients with melanoma are found to have GI tract metastasis at autopsy [3]. According to a review performed at Memorial Sloan Kettering Cancer Center (MSKCC), the incidence of GI tract metastases of melanomas was calculated to be as follows: liver: 68%, small intestine: 58%, colon: 22%, stomach: 20%, duodenum: 12%, rectum: 5%, esophagus 4% and anus 1% [16]. Other studies have validated these findings [17].

### Pathogenesis of primary melanoma of the colon

#### a. **Relation to neural crest cells**

Neural crest cells are found extensively in the intestines, and are believed to have developed from caudal branchial arches during embryogenesis. In vitro, the bowel has been shown to favor the differentiation of these cells into mature melanocytes. However, this effect has not been successfully replicated *in vivo *which explains the absence of mature melanocytes in the intestines. In theory, melanomas can only arise at sites that house either melanocytes or cells that are capable of melanocytic differentation. Hence, it is no surprise that these tumors are predominantly found in the skin. This also explains why they rarely arise in the colon [18].

#### b. **Model of tumor regression**

The presence of potentially metastatic melanomas in the colon with unknown primaries can be explained by the model of tumor regression. A variation in immunologic status, such as infection or pregnancy, can be associated with spontaneous regression of melanomas in their primary sites. In a study of 437 cutaneous melanoma cases, 12.3% of all tumors showed at least partial regression [19]. Histologic findings seen in cases of tumor regression include dermal lymphocytic infiltration with melanophages, vascular proliferation, absence of malignant melanoma cells and reparative fibrosis [20].

#### c. **Ectodermal differentiation**

However, some colonic melanomas are indeed truly primary tumors. The probable genesis of such tumors involves a concept of "ectodermal differentiation" - that ectodermal cells are capable of differentiation into multiple cell lines and may variably migrate into the colon during the embryologic stages to develop into melanocytes [7]. The omphalomesenteric duct may provide one potential route for this transfer of cells [3]. Such a process may also drive the melanoblastic cells from the anal region into the distal colon. Finally, primitive stem cells localized within the gut wall may also give rise to heterotropic melanocytes in the colon [21] and these in turn can give rise to the primary melanoma of the colon. However, despite these theories, the true pathological basis for the occurrence of melanocytes within the colon remains speculative.

### Investigation of melanoma with an unknown primary

Whenever a seemingly primary melanoma is detected in an atypical location such as the colon, it is prudent to conduct a thorough clinical investigation to consider the possibility of metastatic disease.

#### a. **History and physical examination**

A thorough history and comprehensive physical examination are primary tools in this regard. Oculocutaneous melanomas, being the most common primary melanomas, should be excluded first as a matter of common clinical sense. It is possible that a partially regressed tumor maybe found during this exercise and a high index of suspicion should be entertained to completely exclude a malignant lesion. In one study, head and neck and trunk regions were found to be the favored sites for primary melanomas in men while in women, primary melanomas were seen more commonly in the lower extremity [22]. An examination of all the major lymph node groups should be undertaken as the presence of regional lymphadenopathy in a particular area may give a clue to the site of the primary melanoma if anatomic knowledge of the usual routes of lymphatic drainage is applied to the clinical scenario. Also, melanomas can even arise de novo in lymphatic tissue [23]. Gynecologic and abdominal examinations (including rectal examination +/- proctoscopy) should be performed in patients, especially if they have inguinal lymphadenopathy [24]. Otolaryngologic and ophthamologic examinations should be done as well; however the recommendations regarding the performance of these are not uniformly described. For example, Cormier et al recommend that ophthalmologic exam be done specifically in patients with an unknown primary and visceral metastasis to organs such as the liver [24]. In addition to the complete assessment of the skin, any previous skin biopsies should be reviewed [24]. In the history, the patient should be specifically accosted about long standing, newly experienced or recently modified symptoms in a systematic manner as well as relevant familial history about malignancy.

#### b. **Laboratory and radiological investigations**

Baseline laboratory investigations in such patients should include basic hematological parameters, chest x-ray and ultrasound of the abdomen. Bone scan, magnetic resonance imaging (MRI) or computed tomography (CT) scan of the head and either whole body CT maybe also done [24,25]; however, we feel that the performance of these particular investigations should be dictated by the presentation profile of the patient and the investigative discernment of the physician.

#### c. **Role of follow-up surveillance**

An additional point to consider is the role of surveillance in the follow-up of these patients as an unknown primary, although obscure at first, has been reported to manifest clinically later on [26].

### Clinical parameters

#### a. Age

The average age of patients on presentation in our review was calculated to be 60.4 years. This finding was identical to that found in a review of 24 patients of metastatic colonic melanoma [17].

#### b. **Gender**

We did not find any significant gender predilection with 7 patients being male and 5 being female (male to female ratio of 1.4:1). For comparison, the male to female ratio in patients with metastatic melanoma to the colon has been reported as 1.5:1[17].

#### c. **Sites of involvement**

**Figure 1:**
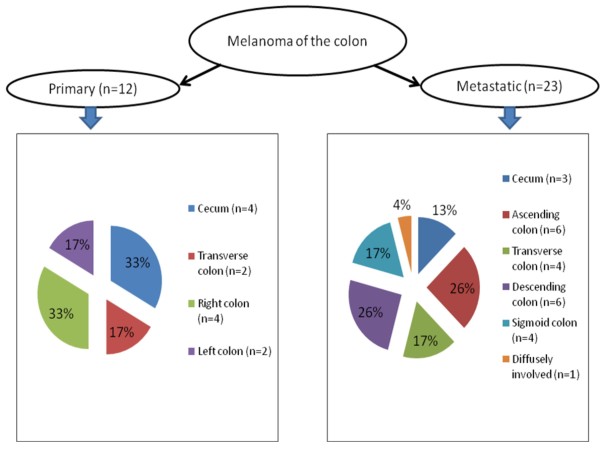
**Comparison of anatomic distribution in primary and metastatic melanoma of colon**.

Right colon and cecum were found to be the most common sites for the occurrence of primary colonic melanomas as depicted in Figure 1. This was slightly different from the data on metastatic colonic melanomas, where ascending colon and descending colon were reported as the predominant sites involved [17]. Figure 1 shows a comparison of the sites involved in primary and metastatic colonic melanoma.

#### d. **Signs and symptoms**

**Figure 2:**
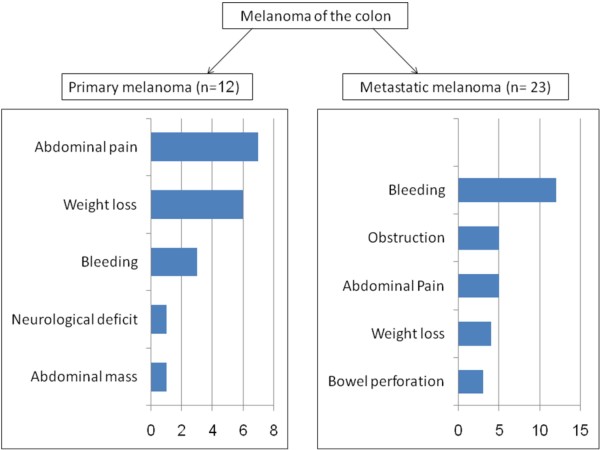
**Comparison of symptoms in patients with primary versus metastatic melanoma of colon**.

In general, metastatic melanoma should be suspected in any patient with a history of melanoma who develops abdominal pain, nausea, vomiting, distension, diarrhea, melena or anemia [10]. In a study of 24 cases of metastatic colonic melanomas at MSKCC, bleeding (50%) was found to be the most common presenting complaint, followed by obstruction (20%), abdominal pain (20%) and weight loss (16%) [17]. Figure 2 shows a comparison of the signs and symptoms seen in primary and metastatic colonic melanoma. Abdominal pain (58%) and weight loss (50%) were the chief presenting complaints of the primary colonic melanoma cases in our review. In contrast, these two complaints were relatively less common in reported cases of metastatic disease [17]. This could be explained by the fact that patients with a history of previously diagnosed melanoma at any location are less likely to present with GI symptoms at the time of diagnosis of colonic melanoma. In addition, they are diagnosed early owing to a higher degree of suspicion and a lower threshold for conducting investigative and confirmatory testing.

### Diagnosis

The diagnostic trajectory of primary colonic melanomas maybe more protracted as compared to their metastatic counterparts owing to the absence of a history of melanoma.

#### a. **Barium studies**

Findings of colonic involvement on barium studies include multiple submucosal nodules, intussusception, large ulcerative lesions, and extrinsic masses compressing the colon [27].

#### b. **Colonoscopy**

Colonoscopy remains the most reliable diagnostic investigation with a high sensitivity and specificity, and offers an additional value of obtaining tissue for diagnosis [17].

In our review, colonoscopy alone was performed in 5 patients for the diagnosis of disease [6,9,12]. Two patients presented with intestinal obstruction secondary to intussusception, so computed tomography (CT) scan alone was performed in them [3,10]. Of the remaining 5 patients, 2 were evaluated with CT scan followed by colonoscopy [4,8] while one each was assessed through CT scan and Barium Enema [5], CT scan and ultrasound of the abdomen [1], and CT scan alone [2]. In patients in whom colonoscopy was not performed, the biopsy specimens required for the tissue diagnosis of melanoma were collected during the surgical procedure. In our review, the average size of resected neoplasms was found to be 5.97 cm in the greatest dimension.

#### c. **Histopathology and immunohistochemistry**

On histopathology, these tumor cells show varying proportions of epitheloid areas and spindle cells. There may be in situ change in the overlying or adjacent GI epithelium, which is identified histologically by the presence of atypical melanocytic cells in the epithelial basal layer and extension in a "pagetoid" fashion into the more superficial epithelium. This feature is reported in 40%-100% of all primary GI melanomas [28]. The tumor cells may either show abundant melanin pigment or may be completely amelanotic [29]. In our analysis of primary colonic melanoma, 14% of the cases were found to be amelanotic, compared to 30% reported in literature for metastatic melanoma [17].

The use of special immunohistochemical stains may prove to be a further aid in securing the diagnosis. S-100 is highly sensitive for diagnosing melanomas, and our results calculated the sensitivity of this marker to be 90%. HMB-45 on the other hand is very specific for the detection of this malignancy. This is because it recognizes a premelanosomal glycoprotein related to the tyrosinase system, and may thus be negative in undifferentiated amelanotic neoplasms [29]. In our review, 100% of the pathologic specimens tested for HMB-45 were positive; making it highly sensitive and specific for tumor diagnosis.

Once the definitive diagnosis of colonic melanoma has been established, the next question that accosts a clinician is the determination of the primary or metastatic origin of the neoplasm. Ozdemir et al proposed strict diagnostic criteria for bronchial melanoma to be classified as a primary lesion. The same may be applied to the colonic lesions as well. According to these criteria, (1) the lesion must be solitary in the surgical specimen (2) there must be no previously excised skin tumor (3) no previous ocular tumor (4) morphology must be compatible with primary tumor (5) there must be no other demonstrable melanomas at the time of surgery (6) findings should be confirmed by careful autopsy [30]. Colonic melanomas not fulfilling these criteria should ideally be termed secondary. In our review, all these criteria were met for all patients except the sixth criterion, i.e. confirmation by autopsy, which was not done in any of our cases.

### Differential diagnosis

There are three other entities that have potentially overlapping clinical manifestations with colonic melanoma. They include gastrointestinal stromal tumor (GIST), clear cell sarcoma of soft parts (CCS), and epithelioid malignant peripheral nerve sheath tumor [31].

#### a. Gastrointestinal Stromal Tumor

On endoscopic evaluation, amelanotic GI melanomas show striking resemblance to GIST [12]. Furthermore, nearly 55% of melanomas show some staining for CD117, and conversely 10% of GISTs stain positively for S100, particularly if there is neural differentiation [31,32]. However, strong S-100, HMB-45 and melan-A positivity and negativity for CD117 favors the diagnosis of melanoma. Colonic GISTs are also uncommon; representing only 5% of all GISTS [33].

#### b. Clear Cell Sarcoma

Another differential to be kept in mind is CCS, which usually harbors the aponeurotic and tendinous areas of the extremities but can rarely involve the viscera. Like melanomas, they may also be S-100 positive, but the histological appearance is usually sufficient to make the correct diagnosis. CCS appears as a monotonous population of round to oval cell with clear cytoplasm and prominent nucleoli [34]. If there are still doubts, the (12;22)(q13;q12) translocation can be used to identify CCS, as demonstrated by Covinsky et al [35].

#### c. Epithelioid malignant peripheral nerve sheath tumor

Lastly, the epithelioid variant of malignant peripheral nerve sheath tumor should also be ruled out. These neoplasms constitute a spindle cell morphologic structure that is usually monotonous in appearance and may exhibit S-100 positivity. Areas of necrosis, large vascular spaces and perivascular concentration of neoplastic cells are additional findings in favor of epitheloid malignant peripheral nerve sheath tumor [36].

### Management

For primary colonic melanomas that have not metastasized to any distant parts of the body, surgical resection with wide margins appears to be the treatment of choice. Chemotherapeutic agents including interferons, cytokines, biological agents and radiation therapy for brain metastases have all been reported as adjuvant and palliative options while considering malignant melanomas in general [8].

#### a. Surgical Management

Aggressive surgical resection has traditionally been the mainstay of treatment for most melanomas that have not disseminated at the time of diagnosis. This may be followed by postoperative radiation therapy, chemotherapy and immunotherapy for any residual disease or nodal involvement [33,37]. Surgical intervention may even be warranted for metastatic melanoma of the gut, since it is not only palliative but also significantly affects the prognosis [37,38]. As many as 80-90% cases with non-curable melanoma of the GI tract are likely to experience symptomatic relief following palliative surgery [17].

A review of literature on primary melanoma of the colon did not reveal any specific criteria that authors have used to assign specific surgical modalities to patients. Since the patients were managed by different surgeons, each management approach was individualized according to the judgment and discernment of the surgeon. However, in general, it is easy to appreciate the general trend that patients with limited, controlled disease were managed with curative surgical therapy.

Nine out of 12 patients underwent surgical intervention in our review; 7 of these cases had undergone hemicolectomies, one had undergone partial colectomy and one case was managed with laparoscopic ileocecal resection. The extent of the colon removed in the partial colectomy was not specified by Mori et al [6]. It appears that traditional hemicolectomy is a favored approach for primary melanoma of the colon in most instances. For most patients, the authors did not mention if lymphadenectomy was performed. Only 1 report explicitly stated that lymphadenectomy was carried out [6].

As data on primary colonic melanoma is limited, we may also consider the results of studies reported in literature for the metastatic melanoma and attempt a logical extrapolation of these to primary colonic melanoma. A study enrolled 24 patients who were diagnosed with metastatic colonic melanoma at Mayo Clinic Rochester, Scottsdale and Jacksonville during the interval 1960-2000 [17]. Seventy five percent of the patients underwent surgical resection of the tumor with nodal sampling. Patients with positive nodes had an average survival of 20.4 months while those with negative nodes lived an average of 34.7 months, with the average being 27.45 months. Overall, the one-year and five-year survival rates were 37% and 21% respectively. Again, the main limitation is that the authors didn't calculate individual survival rates for the type of surgery performed. Furthermore, those patients who underwent postoperative chemotherapy had an average survival of 28.3 months compared to 23.3 months for those who did not, but this difference was not statistically significant [17]; although a five month survival advantage appears to be a clinically significant and intuitively reasonable advantage afforded by postoperative chemotherapy. Signs of bowel obstruction and perforation were related to poor survival, with an average life expectancy of less than 10 months [17]. None of the patients in our review had perforation while two of the patients presented with intestinal obstruction secondary to intusseception. However, after surgical intervention, both of these patients remained recurrence free for atleast 20 months and 36 months respectively. Literature on the subject shows that surgical resection of uncomplicated colonic melanomas has led to better outcomes with one patient surviving to 38 months and the other lost to follow up at 13 months in one series [22].

Of the 12 patients of primary colonic melanomas in our review, 9 underwent surgical resection of the colonic tumor without any recurrence of the disease in their respective follow-ups. The average recurrence free interval was 2.59 years in the 12 cases we reviewed. In our review of existing cases of primary colonic melanoma, only one patient was managed with laparoscopic surgery (resection of ileocecum) [12]. This approach needs to be evaluated in further studies.

#### b. Chemotherapy

Melanoma is generally believed to be a chemotherapy-resistant neoplasm. Nevertheless, several chemotherapeutic agents have shown activity ranging from 10% to 25%. Numerous combination chemotherapy regimens have also been evaluated, but survival benefit is yet to be demonstrated [39]. Furthermore, chemotherapy appears to have a role in only the disseminated cases of primary malignant melanoma. Dacarbazine has remained the standard of care for metastatic melanoma over the last four decades. Temozolamide is another option that was found to have a response rate comparable to that of dacarbazine in a randomized control trial. It offers two potential advantages as it readily crosses the intact blood-brain barrier and can be used as an oral agent. It was also used in one of the patients in our review, who presented with brain metastasis. Other promising options for chemotherapy in melanoma include cisplatin, carboplatin, nitrosoureas, docetaxil and pacetaxil [39].

First described in 1984, Dartmouth regimen (dacarbazine/carmustine/cisplatin/tamoxifen) was the first successful combination therapy used against melanomas, with a response rate of 55% observed in a group of 20 patients [40]. Other successful combination regimens used against melanomas include cisplatin/vinblastine/dacarbazine [41] and carboplatin/paclitaxel [42].

### Prognosis

The prognosis of primary malignant melanoma of the colon appears to be better than other types of primary mucosal melanomas. However, both tend to be more aggressive than their cutaneous counterparts [5]. According to a study on metastatic colonic melanomas, the overall morality was 47%, with one-year and five-year survival rates of 60% and 33% [17]. One could assume the prognosis of the primary colonic melanomas to be comparatively better. In our review, 9 reports mentioned a documented follow-up in their patients. Two of these 9 patients died; hence, the mortality rate was 22.2%.

### Future directions

The past few years have seen numerous advances in our knowledge regarding the cell signaling pathways that propel melanoma in humans. The RAS/RAF/MEK/ERK is one of them, which plays an essential role in melanoma cell growth, invasion, and survival. This has been the focus of avid investigation as a novel therapeutic target with drugs such as Sorefenib. Such agents have also been combined with other chemotherapeutic drugs such as dacarbazine with reasonable results. However, the major limiting factor is that not all melanoma patients have the mutation which is essential for Sorafenib to work [43]. In any case, the emergence of targeted therapies looks very promising for the management of melanoma in the years to come.

## Conclusion

Primary melanoma of the colon is a rare clinical entity in medical literature. Whenever a seemingly primary melanoma is detected in an atypical location such as the colon, it is prudent to conduct a thorough clinical investigation to consider the possibility of metastatic disease. We have presented a review of 12 cases of primary melanoma of the colon that have been reported in the literature so far in order to gain a better understanding of the unique features, symptoms, diagnosis and management of this rare tumor. However, it is acknowledged that further studies are needed to document the long term follow-up, survival advantage and safety profile of the management approaches employed in patients with primary colonic melanoma. Based on a synthesis of the current data, it appears that the management of primary colonic melanomas should focus primarily on surgical resection, unless the disease has metastasized to distant sites where surgery may have a more limited palliative role. Targeted biological therapies are a promising prospect in the management of primary colonic melanoma in the future and should be explored further.

## Competing interests

The authors declare that they have no competing interests.

## Authors' contributions

AMI collected the data, helped in its interpretation and drafted the manuscript. UK and TS performed data acquisition, data analysis, data interpretation, preparation of illustrations and drafted the manuscript. MRK conceived the study, interpreted the data, drafted the manuscript and provided overall supervision in the project. All authors read and approved the final manuscript.
